# Supplementary material for: What Is the Exact Contribution of PITX1 and TBX4 Genes in Clubfoot Development? An Italian Study
Source: Genes (Basel). 2022 Oct 27;13(11):1958. doi: 10.3390/genes13111958 (PMC9690101; doi:10.3390/genes13111958)
Supplement: Supplementary file 1 [file genes-13-01958-s001.zip › genes-1942838-supplementary.pdf]

Table S1. Primer Sequence.

TBX4_1For	GTTCCCTCCTCCAGCTCAG
TBX4_1Rev	CCGCCCTCTCCTCTATCTC
TBX4_2For	CAGCTCTTCGGGTCTGGTTC
TBX4_2Rev	CTTCTAAGGGACCTCCCTG
TBX4_3For	GGGAAGTGAGTTGTGCAGG
TBX4_3Rev	GCAGACATTCGCTGACCAC
TBX4_4For	GACAATGTGGTGGGACCAG
TBX4_4rev	CAGAGGCTTGAGCTCCTTC
TBX4_5For	GAAATGGCTCTGGGAGTGC
TBX4_5Rev	CATTCGATTTGCTCGCGGC
TBX4_6For	GGGTAGTGAGAAGCGGTG
TBX4_6Rev	GCCACACAAGCACGAGATG
TBX4_7For	GAAATGGCTCTGGGAGTGC
TBX4_7Rev	CTTGGTAGAGTGCAGGCC
TBX4_1For	GTTCCCTCCTCCAGCTCAG
TBX4_1Rev	CCGCCCTCTCCTCTATCTC
TBX4_2For	CAGCTCTTCGGGTCTGGTTC
TBX4_2Rev	CTTCTAAGGGACCTCCCTG
TBX4_3For	GGGAAGTGAGTTGTGCAGG
TBX4_3Rev	GCAGACATTCGCTGACCAC
TBX4_4For	GACAATGTGGTGGGACCAG
TBX4_4rev	CAGAGGCTTGAGCTCCTTC
TBX4_5For	GAAATGGCTCTGGGAGTGC
TBX4_5Rev	CATTCGATTTGCTCGCGGC
TBX4_6For	GGGTAGTGAGAAGCGGTG
TBX4_6Rev	GCCACACAAGCACGAGATG
TBX4_7For	GAAATGGCTCTGGGAGTGC
TBX4_7Rev	CTTGGTAGAGTGCAGGCC

PITX1_1For	CGCTGTCGCTTTAAGACAGA
PITX1_1Rev	GGAGAGGGAGCTTGGTTGC
PITX1_2For	CTTTCCAGTGGGGACAGTTC
PITX1_2Rev	GACCTTCAACCCCTTTGACC
PITX1_3For	CAGGAAAGGAACCATTGTGG
PITX1_3Rev	CTGCGCAGGTGTGAGGTC

Table S2. Family’s cases.

cod_pt	Institute	sex	side	DNA	relationship	Family
PT146	RIZZOLI	F		S	proband	Family_1
PT147	RIZZOLI	F		S	proband	
PT144	RIZZOLI	M		S	father of PT146 and PT147	
PT145	RIZZOLI	F		S	mother of PT146 and PT147	
PT129	BURLO	M		N	proband	Family_2
PT56	BURLO	M		S	father of PT83	
PT128	BURLO	F		N	mother of PT83 and PT129 and PT130	
PT130	BURLO	F		N	proband	
PT83	BURLO	F		S	proband	
PT91	RIZZOLI	M	BIL	S	proband	Family_3
PT90	RIZZOLI	M	BIL	S	proband	
PT175	RIZZOLI	M		S	proband	Family_4
PT171	RIZZOLI	F		S	grandma of PT175	
PT170	RIZZOLI	F		S	mother of PT171 and PT172 grandmother of PT175 (sick)	
PT172	RIZZOLI	M		S	great-uncle of PT175 (sick)	
PT173	RIZZOLI	F		S	mother of PT175	
PT174	RIZZOLI	M		S	Father of PT175	
PT25	S.RAFFAELE	M	BIL	S	proband	Family_5
PT26	S. RAFFAELE	M	BIL	S	father of PT25(sick)	
PT31	S.RAFFAELE	F		S	mother (grandma) of PT27 PT28 and PT29	Family_6
PT27	S.RAFFAELE	M	BIL	S	uncle of PT30 (son of PT31)(sick)	
PT28	S.RAFFAELE	M	MONO	S	uncle of PT30 (son of PT31)(sick)	
PT29	S. RAFFAELE	F	BIL	S	ther of PT30(sick) (daughter of PT	
PT30	S.RAFFAELE	M	BIL	S	proband	
PT46	BURLO	M		S	father of PT45	Family_7
PT45	BURLO	M	BIL	S	proband	
PT47	BURLO	F		S	mother of PT45	
PT148	BURLO	M		S	proband	
PT115	RIZZOLI	M		S	proband	Family_8
PT141	RIZZOLI	F		S	proband	
PT55	BURLO	F	L	S	proband	Family_9
PT65	BURLO	F		S	sister of PT55	
PT134	BURLO	M		S	proband	Family_10
PT38	RIZZOLI	M	BIL	S	proband	
PT135	BURLO	M		S	father of PT134 cousin of PT133	
PT133	BURLO	F		S	mother of PT134	
PT94	RIZZOLI	F		S	mother of PT86	Family_11
PT86	RIZZOLI	M	R	S	proband	
PT184	BURLO	M		S	father of PT185	Family_12
PT186	BURLO	F		S	mother of PT185	
PT185	BURLO	M		S	fratello	
PT116	BURLO	M		S	proband	
PT68	BURLO	M		S	padre	Family_13
PT69	BURLO	M		S	proband	
PT41	RIZZOLI	M		S	father of PT40	Family_14
PT40	RIZZOLI	M	L	S	proband	
PT24	SAN RAFFAELE	F		S	mother of PT21 and PT22	Family_15
PT21	SAN RAFFAELE	F	BIL	S	proband	
PT23	SAN RAFFAELE	M		S	father of PT21 and PT22	
PT22	SAN RAFFAELE	F	BIL	S	proband	
PT153	BURLO	M		S	proband	Family_16
PT155	BURLO	M		S	father of PT153 and PT156	
PT150	BURLO	F		S	mother of of PT153 and PT156	
PT156	BURLO	M		S	proband	
PT105	BURLO	M		S	proband	Family_17
PT106	BURLO	M		S	father (sick) of PT105	
PT96	BURLO	M		S	proband	Family_18
PT118	BURLO	M		S	grandfather (sick) ot PT96	

BIL: Bilateral; L: left; R: right; M: male; F: female.

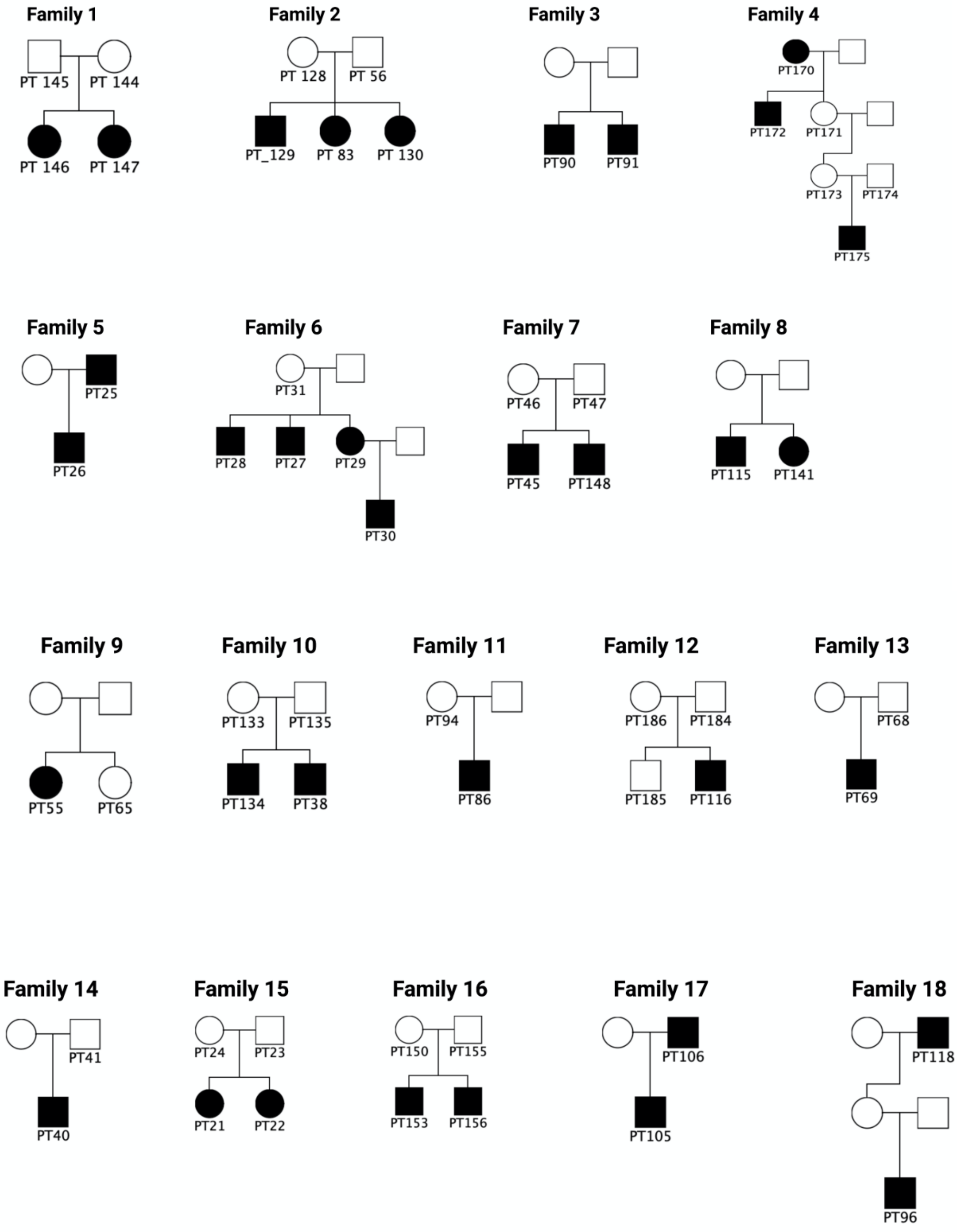

Figure S1. Family's Pedigree listed in Table S2.

Table S3. Isolated probands.

cod_pt	Institute	sex	side	patient
PT1	BURLO	M	BIL	proband
PT2	BURLO	M	BIL	proband
PT3	BURLO	M	BIL	proband
PT4	BURLO	M	BIL	proband
PT5	BURLO	M	R	proband
PT6	BURLO	M	BIL	proband
PT7	BARI	F	BIL	proband
PT8	BARI	F	BIL	proband
PT9	BARI	F	BIL	proband
PT10	BARI	M	L	proband
PT11	BARI	M	BIL	proband
PT12	BURLO	M		proband
PT13	BARI	M	BIL	proband
PT14	BARI	M	L	proband
PT15	BARI	M	BIL	proband
PT16	BARI	M	L	proband
PT17	BARI	M	BIL	proband
PT18	BURLO	F	BIL	proband
PT19	BURLO	M		proband
PT20	BURLO	M	BIL	proband
PT32	BURLO	F	BIL	proband
PT33	BURLO	M	BIL	proband
PT 34	BURLO	F	L	proband
PT35	BURLO	M	L	proband
PT36	RIZZOLI	M	R	proband
PT37	RIZZOLI	M	L	proband
PT39	RIZZOLI	M	BIL	proband
PT42	RIZZOLI	M	R	proband
PT43	BURLO	M	BIL	proband
PT44	BURLO	M	L	proband
PT48	BURLO	F	L	proband
PT49	BURLO	M	R	proband
PT50	BURLO	F	R	proband
PT 51	BURLO	M		proband
PT52	BURLO	M		proband
PT53	BURLO	F		proband
PT54	BURLO	M	L	proband
PT57	BURLO	M		proband
PT58	RIZZOLI	F	R	proband
PT59	RIZZOLI	M	BIL	proband
PT60	RIZZOLI	F	BIL	proband
PT61	RIZZOLI	M	BIL	proband
PT62	RIZZOLI	M	BIL	proband
PT63	RIZZOLI	M	BIL	proband
PT64	BURLO	M		proband
PT66	BURLO	F	BIL	proband
PT67	BURLO	F	BIL	proband
PT70	BURLO	M		proband
PT71	BARI	M	R	proband
PT72	BARI	M	BIL	proband
PT73	BARI	M	L	proband
PT74	BARI	M	BIL	proband
PT75	BARI	F	R	proband
PT76	BARI	M	R	proband
PT77	BARI	F	R	proband
PT78	BURLO	M		proband
PT79	RIZZOLI	M	L	proband
PT80	RIZZOLI	M	L	proband
PT81	RIZZOLI	M	R	proband
PT82	RIZZOLI	M		proband
PT84	RIZZOLI	M	BIL	proband
PT85	RIZZOLI	M	BIL	proband
PT87	RIZZOLI	M	R	proband
PT88	RIZZOLI	M		proband
PT89	RIZZOLI	M	BIL	proband
PT92	RIZZOLI	M	BIL	proband
PT93	RIZZOLI	M	BIL	proband
PT95	RIZZOLI	M		proband
PT97	BURLO	F		proband
PT98	RIZZOLI	M	BIL	proband
PT99	RIZZOLI	M	BIL	proband
PT100	RIZZOLI	M	L	proband
PT101	RIZZOLI	M		proband
PT102	BURLO	M	BIL	proband
PT103	BURLO	M		proband
PT104	BURLO	M	R	proband
PT107	RIZZOLI	M		proband
PT108	RIZZOLI	M		proband
PT109	RIZZOLI	M		proband
PT110	RIZZOLI	M		proband
PT111	RIZZOLI	M		proband
PT112	RIZZOLI	F		proband
PT113	RIZZOLI	F		proband
PT 114	RIZZOLI	M		proband
PT117	BURLO	F		proband
PT119	BUZZI	M		proband
PT120	MILANO	M		proband
PT121	BUZZI	M		proband
PT122	BUZZI	F		proband
PT123	BURLO	M	R	proband
PT 124	BURLO	M	BIL	proband
PT125	BURLO	M	R	proband
PT126	BURLO	M	BIL	proband
PT127	BURLO	F	BIL	proband
PT 131	BURLO	F		proband
PT132	BURLO	M	BIL	proband
PT136	BURLO	F		proband
PT137	BURLO	M	BIL	proband
PT138	RIZZOLI	M		proband
PT139	RIZZOLI	F		proband
PT140	RIZZOLI	M		proband
PT142	RIZZOLI	F		proband
PT143	RIZZOLI	M		proband
PT 149	BURLO	F		proband
PT 151	BURLO	M		proband
PT152	BURLO	M		proband
PT154	BURLO	M		proband
PT157	BURLO	M		proband
PT158	BURLO	F		proband
PT159	BURLO	M	L	proband
PT160	BURLO	M	R	proband
PT161	BURLO	M	L	proband
PT162	BURLO	M	L	proband
PT163	BURLO	M	BIL	proband
PT164	BURLO	M	BIL	proband
PT165	BURLO	M	L	proband
PT166	BURLO	M	BIL	proband
PT167	BURLO	M	BIL	proband
PT168	BURLO	M	BIL	proband
PT169	BURLO	M	BIL	proband
PT176	BURLO	M	R	proband
PT177	BURLO	M	BIL	proband
PT178	BURLO	F		proband
PT179	BURLO	M	BIL	proband
PT180	BURLO	M	L	proband
PT 181	BURLO	M	BIL	proband
PT 182	BURLO	M	R	proband
PT183	BURLO	F	R	proband
PT187	BURLO	F		proband
PT188	BURLO	M		proband
PT189	BURLO	M		proband
PT190	BURLO	M		proband
PT191	BURLO	M		proband
PT192	BURLO	F		proband
PT193	BURLO	M		proband

M: male; F: female; BIL: bilateral; L: left; R: right.

Table S4. *TBX4* SNPs.

rs	Nucleotide Change	Amino acid changes	MAF	Variant	Patients
rs3744448	c. G17C	p. G6A	C=0.1675	non synonymous	PT158,PT66,PT1,PT101,PT11,PT131,PT142,PT147,PT18,PT187,PT190,PT38,PT50,PT54,PT67,PT69,PT7,PT76,PT77,PT79,PT88,PT116,PT119,PT12,PT188,PT20,PT3,PT36,PT53,PT148,PT154,PT159,PT164,PT166,PT181
rs3744447	c.T276G	p.A92A	G=0.1901	synonymous	PT1,PT101,PT11,PT131,PT142,PT147,PT18,PT187,PT190,PT38,PT50, PT54,PT67,PT69,PT7,PT76,PT77,PT79,PT88,PT116,PT119,PT12,PT188,PT20,PT3,PT36,PT53, PT10,PT103,PT120,PT13
rs758596	c.402-8 G>A		A=0.2788	intronic	PT6,PT158,PT66,PT116,PT119,PT12,PT188,PT20,PT3,PT36,PT53,PT169,PT17,PT175,PT176,PT177,PT178,PT179,PT180,PT160,PT162,PT15,PT152, PT121,PT122,PT132,PT14,PT143,PT104,PT111,PT100, PT165,PT169,PT17,PT175,PT176,PT177,PT178,PT179,PT180,PT182,PT30,PT33,PT34,PT40,PT42,PT48,PT5,PT56,PT57,PT63,PT71,PT72,PT74,PT81,PT82,,PT84,PT86,PT91,PT13
rs777880490	c.C24T; p.S8S	p.S8S	T=0.00008714	synonymous	PT6
NO SNP					PT2,PT4,PT8,PT9,PT102,PT105,PT107,PT108,PT109,PT110,PT112,PT113,PT114,PT115,PT117,PT125,PT126,PT127,PT129,PT130,PT134,PT136,PT137,PT138,,PT139,PT140,PT141,PT146,PT149,PT151,PT153,PT156,PT157,PT16,PT183,PT185,PT189,PT191,PT192,PT21,PT22,PT25,PT27,PT28,PT29,PT32,PT35,PT37,PT39,PT43,PT44,PT45,PT49,PT51,PT52,PT55,PT58,PT59,PT60,PT61,PT62,PT64,PT65,PT70,PT73,PT75,PT78,PT80,PT83,PT85,PT87,PT89,PT90,PT92,PT93,PT95,PT96,PT97,PT98,PT99,PT19

Variants identified in *TBX4* gene. All are predicted as benign or likely benign. Three in coding region (Two synonymous and one missense) and one intronic. MAF: Minor Allelic Frequency.
